# SDM transmission of orbital angular momentum mode channels over a multi-ring-core fibre

**DOI:** 10.1515/nanoph-2021-0471

**Published:** 2021-11-19

**Authors:** Jingxing Zhang, Zhongzheng Lin, Jie Liu, Junyi Liu, Zhenrui Lin, Shuqi Mo, Shuqing Lin, Lei Shen, Lei Zhang, Yujie Chen, Xiaobo Lan, Siyuan Yu

**Affiliations:** State Key Laboratory of Optoelectronic Materials and Technologies, School of Electronics and Information Technology, Sun Yat-sen University, Guangzhou 510006, China; State Key Laboratory of Optical Fiber and Cable Manufacture Technology, Yangtze Optical Fiber and Cable Joint Stock Limited Company, Wuhan 430073, China

**Keywords:** fibre optics communications, orbital angular momentum, ring-core fibre, space-division multiplexing

## Abstract

Spatial division multiplexed optical transmission over a multi-ring-core orbital angular momentum (OAM) fibre is reported for the first time. The seven cores in the fibre each supports OAM modes belonging to mode groups (MGs) of topological charge |*l*| = 0–4. The MGs of |*l*| = 1–4 each contains four near-degenerate OAM modes that carry the combinations of opposite orbital and spin angular momenta. The weak coupling between these higher-order MGs as well as between the cores enables the simultaneous transmission of 56 OAM mode channels (two MGs per core of the topological charges |*l*| = 2 and 3) over the 60-km span, while only requiring modular 4 × 4 multi-input multi-output (MIMO) signal processing to equalize the mixing among the four mode channels in each MG that are strongly coupled – a feature that also minimizes the number of filter taps. The mode channels are launched using seven-core single-mode fibre fan-in devices, with the light in all seven cores converted into OAM modes via specially designed plates that carry seven off-axis-compensated phase masks matching the hexagonal configuration of the multi-core fibres. Each mode channel carries 10 WDM wavelengths, equivalently aggregating to a capacity of 31.4 Tbit/s (net 25.1 Tb/s) and a spectral efficiency (SE) of 62.7 bit/s/Hz (net 50.2 bit/s/Hz) with 28-GBaud QPSK modulation per data channel.

## 1 Introduction

As the current single-mode fibres (SMFs) start to encounter bottlenecks in further improving transmission capacity and cost per bit, space-division multiplexing (SDM) that explores the spatial degree of freedom in optical fibres has been intensively studied over the past decade, showing promising potentials as a solution for future fibre-optic communication systems [1]. SDM implementations mainly include mode-division multiplexing (MDM) schemes utilizing few-mode fibres (FMFs) [2, 3] and core-multiplexing schemes employing multi-core fibres (MCFs) [4], [5], [6], both of which can dramatically increase the number of spatial channels in one single fibre. Combinations of these two approaches based on few-mode multi-core fibres (FM-MCFs) can further increase the spatial channel counts per fibre whilst attempting to avoid the need for increasingly large-scale multi-input multi-output (MIMO) processing for MDM schemes.

Recent progress of SDM transmission systems employing FM-MCFs is summarized in Table 1 [7], [8], [9], [10], [11], [12]. Considering the complexity of the MIMO equalization that deals with the inter-spatial-channel crosstalk, low coupling among fibre cores should be ensured to avoid the inter-core MIMO compensation, while in each core only a few modes are used to suppress the MIMO complexity for inter-mode crosstalk compensation. As illustrated in Table 1, the core × mode counts of the FM-MCFs increased from 12 (four cores × three modes [7]) to 36 (12 cores × 3 modes [9]) and then around 100 by utilizing a 36-core three-mode fibre [10] or 19-core six-mode fibres [11]. Such SDM schemes with high number of multiplexed spatial channels are realized at the cost of the large fibre cladding diameters normally more than 200 μm or even up to 440 μm [13], as small core pitch could result in large inter-core crosstalk. In practice, large cladding diameters (especially more than 250 µm) will deteriorate the mechanical stability or lifetimes of the optical fibres [14]. New solutions are desired to balance the spatial channel counts, fibre cladding diameters and the MIMO complexity. Other challenges include the difficulty in equalizing optical gain for all spatial or mode channels in a FM-MCF erbium doped fibre amplifier (EDFA), and the need to create large-scale multi-core multi-mode spatial (de)multiplexers (DEMUX) with high performance.

**Table 1:** Comparison of the reported transmission systems based on multi-core few-mode fibres. All mode counts should be multiplied by 2 to include the two polarization modes.

Ref.	Core count × mode count	Fibre mode utilized	Cladding diameter (μm)	Distance (km)	MIMO complexity	Net SE (bit/s/Hz)	SE-distance product [(bit/s/Hz) km]
[7]	4 × 3	LP modes^a^	160	3.37	6 × 6, 700 taps	130.3	439
[8]	7 × 3	LP modes	192	1	6 × 6	80	80
[9]	12 × 3	LP modes	230	527	6 × 6, 128 taps	94.32	49,706
[10]	36 × 3	LP modes	306	5.5	6 × 6, 201 taps	100.9	555
[11]	19 × 6	LP modes	267	11.3	12 × 12, 200–300 taps	1099.9	12,429
[12]	38 × 3	LP modes	312	13	6 × 6, 249 taps	1158.7	15,063
This work	7 × 4	OAM modes	180	60	4 × 4, 25 taps	50.2	3010

^a^LP modes: linearly polarized modes.

As an alternative to the FMF-based MDM schemes, ring-core fibre (RCF) based orbital angular momentum (OAM) multiplexing transmission systems have been investigated in recent years, which offer advantages in several key aspects, including DSP complexity, realization of the mode (de)multiplexing and optical amplification [15].

The RCF confines light propagation within the ring-core region. In the radial dimension, with higher-order modes suppressed and only first-order OAM modes retained, the number of modes in all high-order OAM MGs with topological charge |*l*| > 0 is constant at four. In order to reduce the MIMO complexity of the RCF-based OAM multiplexing transmission systems, there are two main different approaches to the RCF design. The first is to sufficiently enlarge the effective refractive index difference (Δ*n*_eff_) between the spin–orbital aligned and the spin–orbital anti-aligned OAM mode sub-groups in the same MG, with each sub-group carrying two modes orthogonal in both spin and orbital. Therefore, only 2 × 2 MIMO (as already implemented in the SMF transmission systems), or even no MIMO, can be implemented in such systems. This approach has been shown to be applicable in short-distance transmission systems normally within several kilometres [16], [17], [18]. The other approach attempts to achieve weak coupling between OAM MGs to a sufficiently low level at which relatively long-distance fibre transmission with tens or even hundreds of kilometres could be implemented [3, 19], [20], [21]. It thereby becomes feasible that only 4 × 4 MIMO is used to compensate the crosstalk among the four mode channels within each MG without having to deal with the inter-MG crosstalk. MIMO-free multiplexing can also be implemented using each MG as one channel at the cost of four times fewer number of channels.

In addition, the highly overlapping intensity distribution of the OAM modes in the RCF also helps to achieve equalised optical amplification for all modes [22, 23]. Since there is no radial higher-order mode, the (de)multiplexing for OAM modes in the RCF can be efficiently achieved with coordinate transformation devices [20, 24, 25].

With these significant merits, OAM fibre optic communication has been extensively investigated over the years [15, 26], with the number of OAM modes carried, the data capacity and the transmission distance all been significantly increased (see in ref. [3]). However, to the best of our knowledge, all OAM-MDM transmission experiments reported so far are limited to fibres with one core. SDM transmissions based on multi-core OAM fibres, aiming at further increasing the OAM channel counts and thus the transmission capacity and spectral efficiency per fibre, have not yet been reported, despite the proposition and numerical simulation of a multi-core OAM fibre [27], [28], [29].

In this paper, the experimental demonstration of multi-core SDM/OAM-MDM per core/wavelength-division multiplexed (WDM) per mode transmission is reported for the first time. Over a 60-km specially designed seven-core RCF with a cladding diameter of 180 µm, a total of 56 OAM channels (seven cores × four OAM modes × two orthogonal polarizations) each carrying 10 WDM channels have been successfully transmitted. With 28-GBaud QPSK modulation per data channel, the system has equivalently achieved an aggregate capacity of 31.4 Tbit/s (net 25.1 Tb/s) and a spectral efficiency (SE) of 62.7 bit/s/Hz (net 50.2 bit/s/Hz) with the bit-error rate (BER) below the 20% soft decision forward error correction (FEC) threshold of 2.4 × 10^−2^. The weak coupling among fibre cores and amongst the non-degenerate OAM mode groups (MGs) within each core has been simultaneously realized, thus the system only requires 4 × 4 modular MIMO processing with time-domain equalization tap number of 25 to deal with the near-degenerate intra-MG mode coupling, achieving significant up-scaling of transmission capacity with ultra-low MIMO complexity and reasonably small fibre cladding diameter.

## 2 Fibre design, fabrication and characterization

The design of the multi-ring-core fibre (MRCF) aims to limit the cladding diameter to ≤180 µm to ensure relatively long fibre-span length as well as good optical fibre (or cable) lifetime and mechanical stability. Considering the cores have relatively large effective area (*A*_eff_), the outer cladding thickness (the distance between the centre of the outer cores and the cladding edge) is set to be ≥40 µm to reduce the micro-bending loss and excess loss of the outer cores [13]. The core pitch is set to be ≥45 µm to maintain low inter-core crosstalk (detailed simulation analysis can be found in Supplementary S1). As a result, a hexagonally packed seven-core fibre structure is selected to satisfy all of the above conditions.

**Figure 1:**
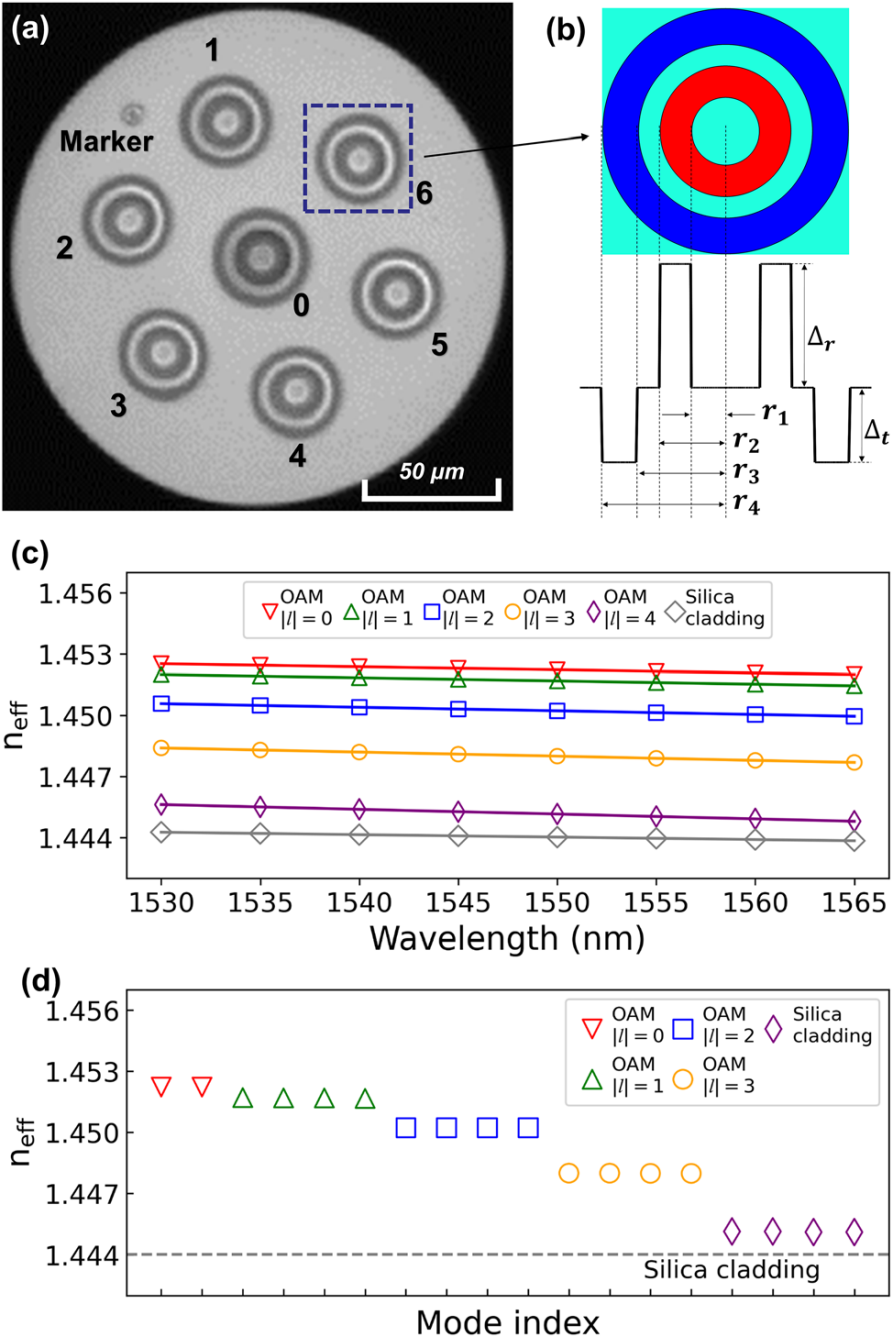
The design of the MRCF. (a) The cross-section of fabricated seven-core RCF; (b) The designed RIP of the ring-core. All the cores have the same RIP. (c) Simulated effective refractive index as a function of wavelength based on the designed RIP. (d) The *n*_eff_ of each mode at 1550 nm, where the markers of the same style represent the *n*_eff_ of different modes in the same MG.

As shown in Figure 1(b), an identical step-index ring-core refractive index profile (RIP) is designed for all fibre cores, whose maximum core-cladding relative RI difference (Δ_*r*_) is set to 0.825% to ensure relatively low fibre-core dopant concentration during the fibre fabrication and thus low fibre attenuation. The inner and outer radii of the ring-core are selected to 4.7 and 9.1 µm, respectively, so that each core supports five OAM MGs (with a topological charge of |*l*| = 0–4) with single radial order. The effective refractive indices of all guided modes within C band of optical wavelengths (from 1530 to 1565 nm) are calculated using a commercial finite element module (COMSOL Multiphysics) based on the designed RIP. As plotted in Figure 1(c), the Δ*n*_eff_ between adjacent OAM MGs with topological charge |*l*| ≥ 1 is greater than 1.5 × 10^−3^, while the four modes in each MG (+/− *l*, each carrying two orthogonal polarizations) are highly degenerate [see inset in Figure 1(d)]. Such a design is aimed at maintaining weak coupling between high-order MGs and strong coupling among intra-MG modes [30, 31]. In addition, a 5-µm wide trench with $\Delta_{t}=-0.5\%$ is introduced around the ring core to confine mode field spreading, which is beneficial to reduce both the inter-core crosstalk and fibre bending loss [32, 33]. All the structure parameters of the seven-core RCF are summarized in Table 2.

**Table 2:** Structural parameters of designed seven-core RCF.

*r*_1_, *r*_2_, *r*_3_, *r*_4_	μm	4.7, 9.1, 12.1, 17.1
Δ_*r*_, Δ_*t*_	%	0.825, −0.5
*n*_*c *_(at 1550 nm)		1.444
Core pitch	μm	50
Cladding diameter	μm	180

The designed seven-core RCF is fabricated under a standard fibre fabrication process and the cross section of the fabricated seven-core RCF is shown in Figure 1(a). The core rods of the seven-core RCF preform are manufactured by a plasma chemical vapour deposition (PCVD) technique. A hole-drilling method is used to obtain the multi-core cylinder, whose internal surface is polished after drilling, ensuring a surface roughness of 50–500 nm. The seven ring-core rods are then inserted into the multi-core cylinder separately to form the fibre preform, which is subsequently drawn into the fibre with a diameter of 180 µm in a standard drawing tower.

**Figure 2:**
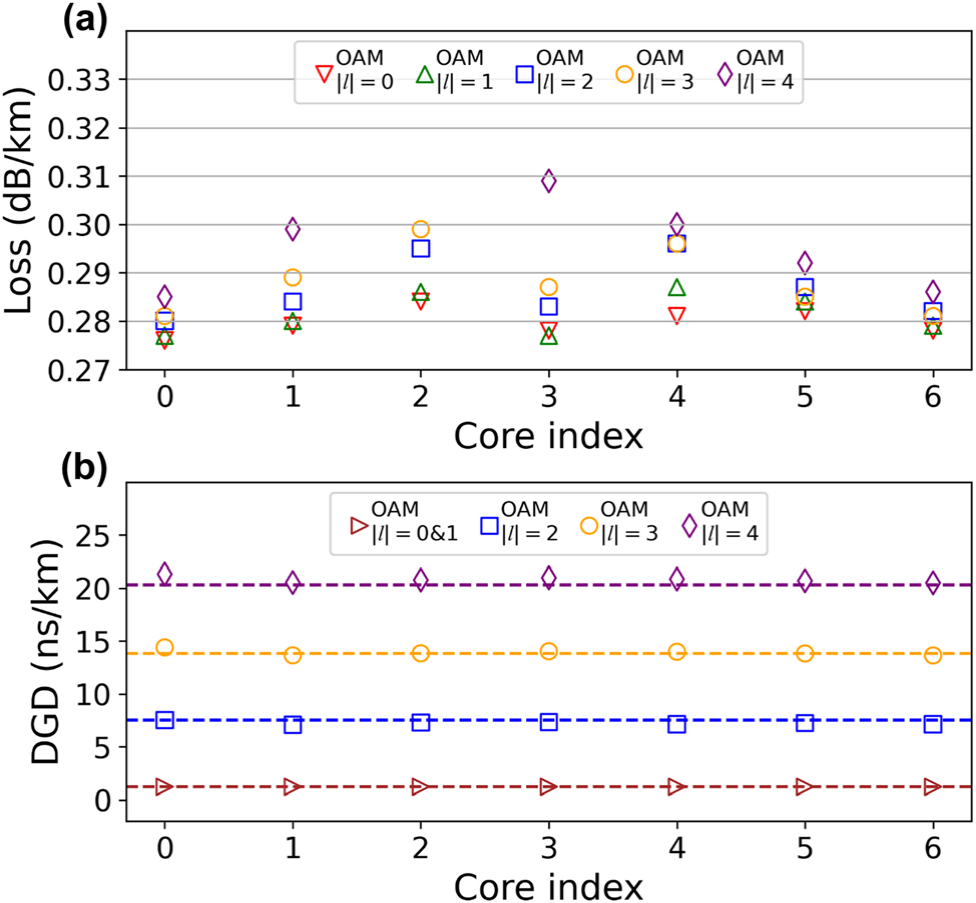
The characterization of the fabricated seven-core RCF. (a) Measured propagation loss of all MGs at 1550 nm along with the fabricated seven-core RCF. The loss of OAM MG |*l*| = 4 for core 2 is 0.36 dB/km and is much higher than other MGs due to the fibre fabrication errors, and is dismissed in this figure. (b) Measured differential group delays (DGDs) of all guided modes of the fabricated seven-core RCF at 1550 nm. Dashed lines represent the simulated results.

The attenuation of all MGs at 1550 nm along the fabricated seven-core RCF is measured utilizing an optical time domain reflectometer (OTDR), with the results summarized in Figure 2(a). Details of the attenuation measurement can be found in Supplementary S2. There is a ∼0.02 dB/km loss difference among different MGs/fibre cores, especially for the highest order OAM MG with topological charge |*l*| = 4, which could be improved by decreasing the optical fibre winding stress and increasing the uniformity of the fibre cores. Such MG/core dependent loss has little consequence in the way of deteriorating the performance of the MIMO equalization, as only modular 4 × 4 MIMO equalization is required to deal with the intra-MG mode crosstalk – the four modes in the same MG are strongly coupled and have almost no differential loss [3].

The differential mode-group delay (DGD) values at the wavelength of 1550 nm are measured based on the time-domain impulse response method using vector network analyser (VNA) [34], as shown in Figure 2(b). Details of the DGD measurement can be found in Supplementary S3. Here we note that since strong coupling occurs between OAM MG |*l*| = 0 & 1 after 60-km transmission, the impulse response of these two MGs merge into one centre of a Gaussian distribution [34] and only an averaged DGD value of OAM MGs |*l*| = 0 & 1 in each fibre core is given for both of the measured and calculated results shown in Figure 2(b).

## 3 Simultaneous spatial and mode multiplexing

The spatial and mode division multiplexing in the seven-core MRCF transmission is realized using free-space optics, in order to achieve weak inter-core and inter-MG coupling simultaneously. Firstly, a ‘7-to-7’ scheme is proposed to excite one specific OAM mode in the seven-core RCF, which is described in Part A of this section. Based on such a scheme, a setup for multiplexing multiple OAM modes in the seven-core RCF is described in Part B. Finally, the scalability and integration of the proposed SDM multiplexing scheme are discussed in Part C.

### 3.1 The ‘7-to-7’ scheme for precise OAM mode excitation in the seven-core RCF

**Figure 3:**
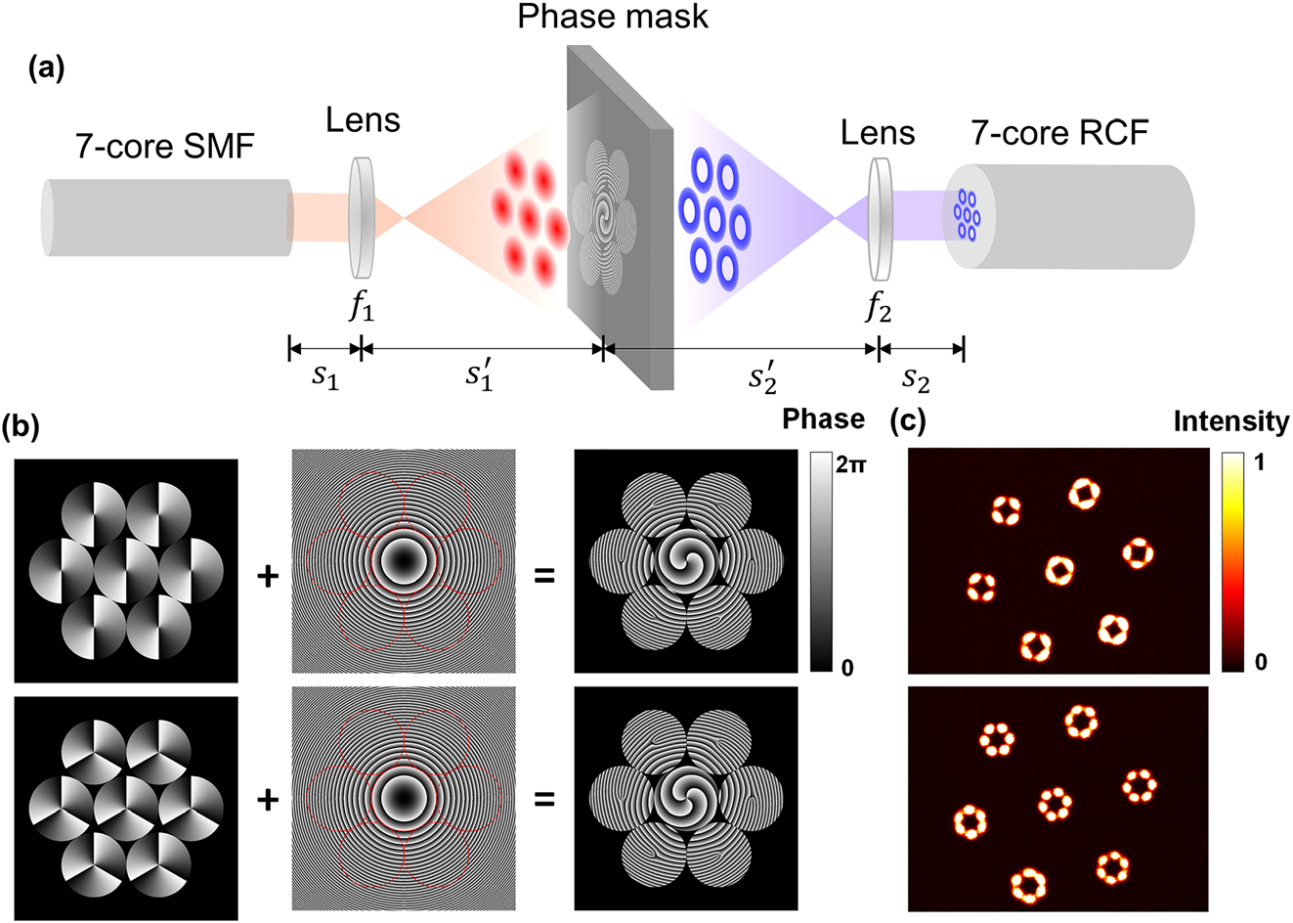
The principle of the proposed '7-to-7' coupling scheme. (a) Schematic diagram of the proposed ‘7-to-7’ scheme for one OAM mode excitation in the seven-core RCF. (b) Hexagonally configured vortex phase plate (VPP) diagram (topological charge of *l* = 2 for the upper and 3 for the lower) are superimposed with a radial quadratic phase distribution. (c) Intensity profile of the OAM modes in the OAM MG with topological charge |*l*| = 2 and |*l*| = 3 after 60-km transmission in the seven-core RCF. Here noted that a LP-mode-like intensity profile in each fibre core is formed by the coherent superposition of four intra-MG OAM modes due to strong coupling among them, even though only one OAM mode is excited in each core at the start of the fibre, and the clear azimuthal distributions of the received intensity profiles of each fibre core still indicate low inter-MG coupling.

Figure 3(a) illustrates the schematic diagram of the proposed ‘7-to-7’ scheme for precise OAM mode excitation in the seven-core RCF. Seven fundamental-mode beams (or Gaussian beams) are emitted from a seven-core SMF [YOFC MCF 7–42/150/250(SM)] connected with a commercial SMF pigtailed fan-in fan-out module (YOFC FAN-7–42), which also has a hexagonal arrangement and thus enables core-to-core mapping to the seven-core RCF. After being imaged onto a phase-only modulation mask by a single thin lens, the seven Gaussian beams are converted to seven OAM beams with the same topological charge and then coupled into the seven-core RCF. Considering the reciprocity of optics, the ‘backward propagation’ from the seven-core RCF to the seven-core SMF in the ‘7-to-7’ system should allow the inverse conversion.

In this process, two key points should be emphasized to ensure efficient and precise mode excitation in each ring core. Firstly, the real amplitude distribution (practically intensity distribution in experiment) of the forward propagating beams and the backward propagating beams should maximally overlap on the phase mask to improve the coupling efficiency (Supplementary S4). Secondly, the phase masks should be designed to compensate the phase mismatch between the forward and backward propagating beams on its plane.

To achieve maximum intensity overlapping, the pitch, beam size and the azimuthal rotation of the forward and backward propagating hexagonally arrayed beams should precisely match on the plane of the phase mask. The rotational matching can be realized by holding the elements on rotating mounts. Here we mainly focus on core pitch and beam size matching on the phase mask between the forward and backward propagating beams. According to thin lens imaging equation [35], the image of the core pitch on the phase-mask plane can be controlled by selecting lens with proper focal length and adjusting the relative positions of the optical fibre end facet, the lens and the phase mask, which satisfy:

(1) $$\frac{1}{s_{i}}+\frac{1}{s_{i}^{\prime }}=\frac{1}{f_{i}}$$

(2) $$V_{i}=-\frac{s_{i}^{\prime }}{s_{i}}$$

where *s*_*i*_ is the distance between the optical fibre end facet and the lens, $s_{i}^{\prime }$ is the distance between the phase mask and the lens, and *f*_*i*_ refers to the focal length of the lens. Here the subscript *i*, whose value equals to 1 or 2, denotes parameters on the seven-core SMF side or seven-core RCF side, respectively, as shown in Figure 3(a). With scaling factor of *V*_*i*_ between the imaging plane and the objective plane, the image of the core pitch *p*_*i*_ becomes $V_{i}\cdot p_{i}$ on the phase-mask plane. With $V_{1}p_{1}=V_{2}p_{2}$ , the image of the core pitch of the seven-core SMF on the phase mask can be well matched with that of the seven-core RCF. As for the beam size matching on the phase mask, it can be easily achieved after the step of core-pitch matching, if the ratio of beam diameter to core pitch of the seven-core SMF is very similar with that of the seven-core RCF. Here in our experimental system, this ratio in the utilized seven-core SMF is slightly smaller than that in the seven-core RCF, as shown in Table 3 that lists the detailed parameters of the elements used in the experimental ‘7-to-7’ setup. Such mismatch can be solved by making the seven-core SMF slightly deviate from the objective plane of the lens and thus increasing the ratio of its beam waist to core pitch. Detailed principle can be found in Supplementary S5.

**Table 3:** Experimental parameters of the ‘7-to-7’ coupling scheme.

Seven-core SMF	Pitch *p*_1_	μm	42
	Mode field diameter *d*_1_	μm	9.5
	*d*_1_/*p*_1_		0.23
	Focal distance *f*_1_	mm	8
	Scaling factor *V*_1_		62
Seven-core RCF	Pitch *p*_2_	μm	50
	Mode field diameter *d*_2_	μm	13.7 (|*l*| = 2), 14.3 (|*l*| = 3)
	*d*_2_/*p*_2_		0.27 (|*l*| = 2), 0.29 (|*l*| = 3)
	Focal distance *f*_2_	mm	8
	Scaling factor *V*_2_		52
Phase mask	Vortex order		+2, −2, +3, −3
	Pitch of vortex phase	μm	2600
	Focal length of the Fresnel lens	mm	226

Phase matching on the phase-mask plane between the two beams is another issue that should be considered. As shown in Figure 3(b), a hexagonally distributed seven-core vortex phase diagram is superimposed with a radial quadratic phase distribution (i.e. Fresnel lens) on the phase mask. The former is utilized to convert the Gaussian beams into the OAM beams with a specific topological charge, while the latter is employed to converge the initially divergent beams [red beams in Figure 3(a)] to match the optical axes of the light beams on the seven-core RCF side of the phase mask [blue beams in Figure 3(a)]. Key parameters of the phase mask used in the ‘7-to-7’ experimental setup can be found in Table 3, and the calculation details are described in Supplementary S5.

After intensity overlapping and phase matching on the phase mask plane, one specific OAM mode can be precisely excited in each core. Figure 3(c) shows the intensity profile of the excited OAM modes in the OAM MG with topological charges |*l*| = 2 and |*l*| = 3 after 60-km transmission in the seven-core RCF. Here we note that a linear-polarization-mode-like intensity profile in each fibre core is formed by the coherent superposition of four intra-MG OAM modes [15], which are generated after long-distance fibre transmission due to strong coupling among these near-degenerate intra-MG modes, even though only one OAM mode is excited in each core at the start of the fibre. However, the clear azimuthal distributions of the received intensity profiles of each fibre core still indicate low inter-MG coupling.

**Figure 4:**
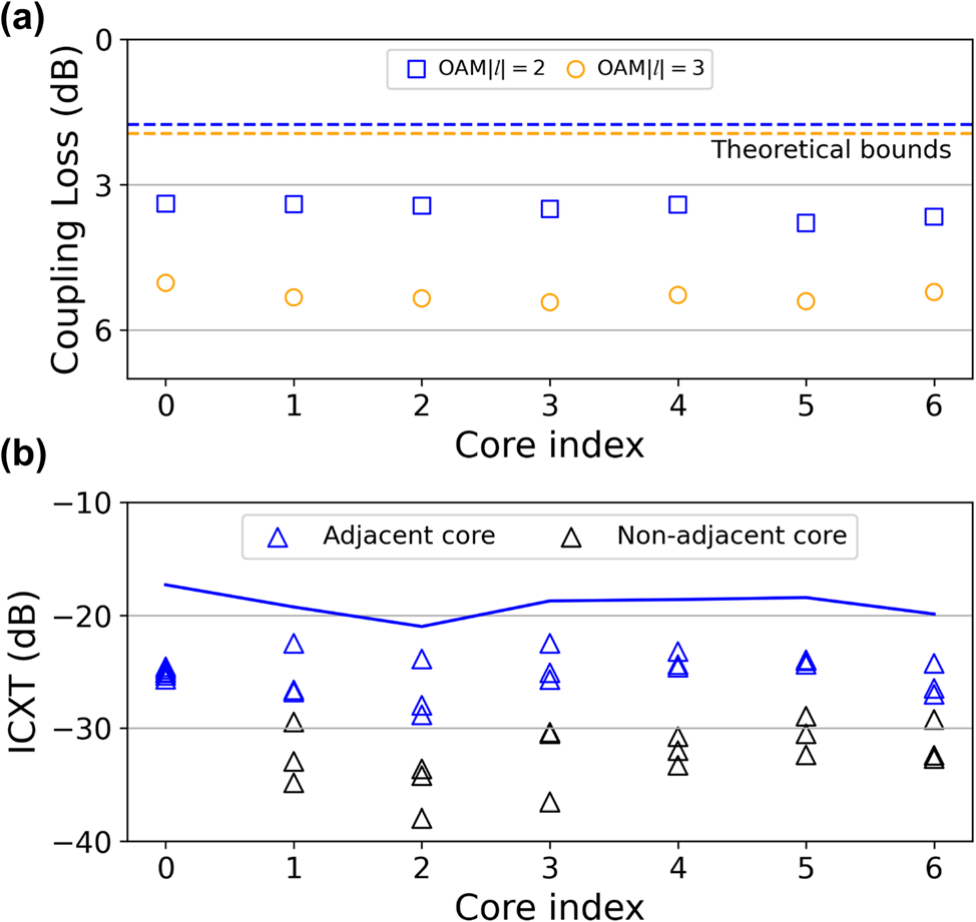
The characterization of the proposed '7-to-7' coupling scheme. (a) Measured insertion loss over seven cores. Dashed lines represent the theoretical lowest coupling loss. (b) Measured inter-core crosstalk. The blue solid line represents the aggregated inter-core crosstalk.

The coupling loss and inter-core crosstalk (ICXT) of the proposed ‘7-to-7’ coupling scheme are characterized, as shown in Figure 4(a) and (b), respectively. The average coupling loss is 3.5 dB for OAM MG |*l*| = 2 and 5.3 dB for OAM MG |*l*| = 3. The average ICXT is −25 dB for adjacent cores and −30 dB for non-adjacent cores.

### 3.2 OAM mode multiplexing in the seven-core RCF

**Figure 5:**
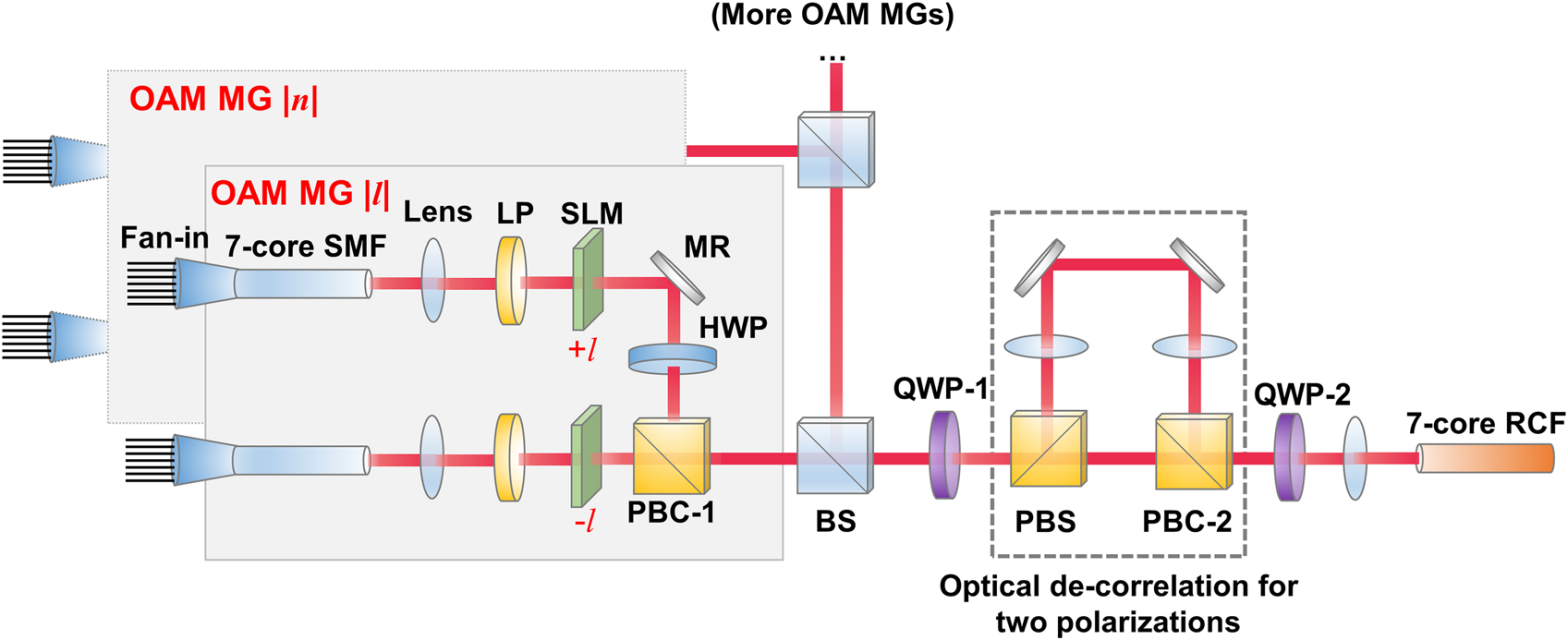
Experimental setup of the seven-core multi-mode multiplexing system. Multiple ‘7-to-7’ branches are used to excite different OAM modes and are combined by the beam splitters. The setup is scalable by constructing more ‘7-to-7’ branches. A polarization multiplexing module is constructed to optical de-correlate for two orthogonal polarizations. SMF, single-mode fibre; LP, linear polarizer; SLM, spatial light modulator; MR, mirror; HWP, half-wave plate; QWP, quarter-wave plate; BS, beam splitter; PBS (PBC), polarization beam splitter (combiner).

Based on the proposed ‘7-to-7’ coupling scheme for launching one OAM mode into each fibre core, mode multiplexing in the seven-core RCF can be achieved by power combining multiple such ‘7-to-7’ branches. Figure 5 illustrates the experimental setup of the seven-core multi-mode multiplexing system. In the module for multiplexing the four intra-MG modes within an OAM MG |*l*| shown in Figure 5, optical beams from one seven-core SMF are imaged to the spatial light modulator (SLM) with a linear polarizer in between, and then converted into OAM beams with topological charge +*l* by the phase mask in the ‘7-to-7’ scheme implemented on the SLM. Meanwhile, another bunch of Gaussian beams are converted into OAM beams with topological charge –*l* through the other ‘7-to-7’ branch in the OAM-MG |*l*| multiplexing module. The two bunches of OAM beams generated are converted into two orthogonal polarizations, respectively, by inserting a half-wave plate (HWP) in one of the branches and combined by a polarization beam combiner (PBC). Thus seven beams each carrying two OAM modes <+*l*, *X*> and <−*l*, *Y*> are created, with *X* and *Y* representing horizontal and vertical polarization, respectively.

Then the generated linearly polarized OAM beams are converted into circular polarizations using a quarter-wave plate (QWP) and injected into the polarization multiplexing module, which consists of a polarization beam splitter (PBS), an optical de-correlation path with a 4-*f* configuration and a PBC, as shown in Figure 5. The PBS is utilized to split the circularly polarized beams into two orthogonal linearly polarized beams with equal power. The de-correlation optical path is in a 4-*f* configuration to keep the pitch and optical axes unchanged for beam-combining. In our experiment, the focal distance of the lenses is chosen to be 100 mm, and the module provides a path length difference of 400 mm between two polarizations, corresponding to a time delay of 1.33 ns or a symbol delay of 37 at a symbol rate of 28 GBaud. After passing through PBC-2 and QWP-2, the 7 optical beams each carrying four OAM modes <+*l*, *R*>, <+*l*, *L*>, <−*l*, *R*> and <−*l*, *L*> are coupled into the seven-core RCF, where *R* and *L* refer to the left and right circular polarization, respectively.

Multiplexing of multiple OAM MGs is realized by power combining more OAM-MG multiplexing modules before the polarization multiplexing module, as illustrated in Figure 5. In this OAM-SDM–WDM transmission experiment, OAM MGs with topological charge |*l*| = 2 & 3 are selected (see Section 4). Taking into account the beam splitter and polarization multiplexing module, there will be extra 6 dB loss for each channel, thus the total multiplexing loss becomes 9.5 dB for channels in OAM MG |*l*| = 2 and 11.3 dB for channels in OAM MG |*l*| = 3.

### 3.3 Discussion on the multiplexing

Simultaneous inter-core and inter-MG weak coupling is realized through proposed ‘7-to-7’ coupling scheme. Compared with the coupling scheme that the cores of a multi-core fibre are mapped to a series of single-core SMF [10], the FIFO-integrated seven-core SMF and seven-core VPPs greatly simplify the optical setup for coupling to a seven-core fibre. With reduced elements, the stability of the setup is enhanced. Robustness to the experimental setup error is analysed in Supplementary S6. Furthermore, the optical setup could be solidified and integrated as a passive multiplexer. For future fibres with more cores (e.g., 19 cores) and/or different core lattice (e.g., square rather than hexagonal), the fan-in integrated SMF needs to be upgraded to the same core number and/or lattice.

Currently the mode multiplexing is based on power combining. With the two MGs used in this work, the power combining loss is still acceptable. This loss will scale up with the number of multiplexed MGs, and advanced multi-core spiral transformation [36] or multi-plane light conversion [37] might be more power efficient solutions for higher number of MGs, which will however require input fibre arrays with complicated 2D lattices.

## 4 OAM-SDM–WDM data transmission

### 4.1 Experimental setup

**Figure 6:**
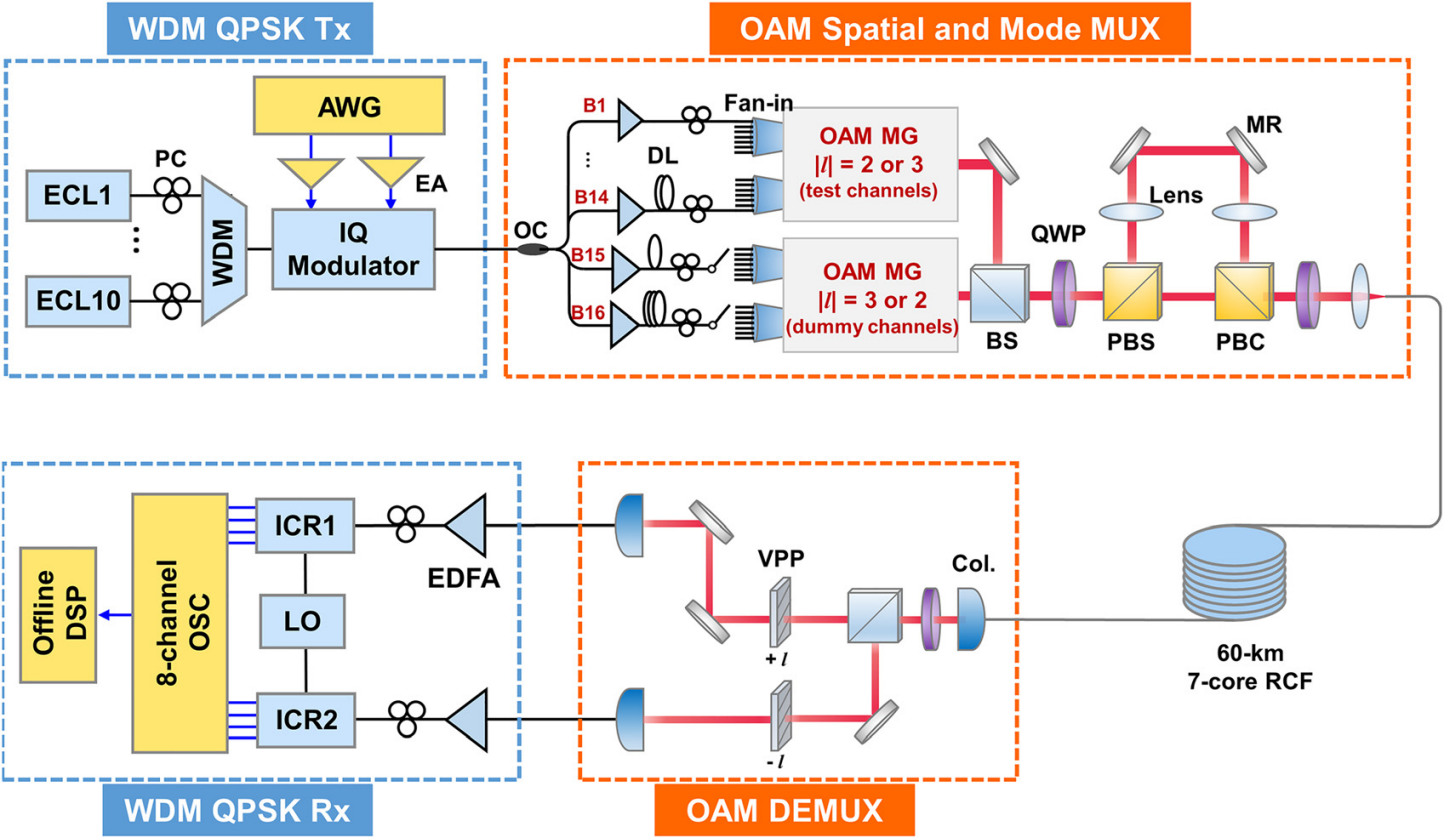
The experimental setup of the OAM-SDM–WDM data transmission system. WDM, wavelength division multiplexing, QPSK, quadrature phase shift keying; AWG, arbitrary waveform generator; ECL, external cavity laser; EA, electric amplifier; OC, optical coupler; OAM, orbital angular momentum; MG, mode group; BS, beam splitter; MR, mirror; QWP, quarter-wave plate; PBS (PBC), polarization beam splitter (combiner). Col., collimator; RCF, ring-core fibre; VPP, vortex phase plate; EDFA, Erbium doped fibre amplifier; ICR, integrated coherent receiver; LO, local oscillator; OSC, oscilloscope; DSP, digital signal processing.

The setup of the OAM-SDM–WDM data transmission experiment is shown in Figure 6. Considering the relatively large Δ*n*_eff_ and thus low inter-MG coupling as well as low fibre attenuation of OAM MGs |*l*| = 2 & 3 (refer to the characterization results in Section 2), these two MGs are selected in the OAM-SDM–WDM transmission system to ensure reasonable optical signal to noise ratio (OSNR) and power budget in the data transmission experiment. At the transmitter, 10 optical carriers from external cavity lasers (ECLs) are combined by a wavelength division multiplexer, with wavelengths on a 0.4 nm/50 GHz grid ranging from 1548.52 to 1552.12 nm. The 10 WDM carriers are then modulated by a 28-GBaud QPSK signal from a two-channel arbitrary waveform generator (AWG) through an in-phase/quadrature (*I*/*Q*) modulator. The sampling rate of the digital-to-analog converter (DAC) of the AWG is set to 56 GSa/s and the data sequence of the generated QPSK signal is pseudo-random binary sequence (PRBS) with a pattern length of 2^18^−1. Here we note that the adjacent WDM carriers are modulated with the same electronic signal due to the hardware limitation in our lab, which will have little impact on the system performance when sufficient protection intervals (best no less than 20% wavelength channel) exist between adjacent WDM channels [38].

Subsequently, the WDM signals are split into 16 branches, which are separately delayed for signal de-correlation and then divided into two groups. The first group includes 14 branches (B1 to B14 in Figure 6), which are utilized as the test channels and connected to the 14 input ports of the first seven-core OAM-MG multiplexing module (details in Figure 5) one by one, to excite test MGs in all seven cores. After passing through the polarization multiplexing module with the 4-*f* de-correlation path (details can be found in Part B, Section 3), the 28 OAM channels (seven cores × two intra-MG OAM modes × two orthogonal polarizations) each carrying 10 WDM channels are generated for test. Meanwhile, the other two branches in the second group (B15 and B16 in Figure 6), that are finally converted to four intra-MG OAM mode channels (one core × two intra-MG OAM modes × two orthogonal polarizations), are utilized as the dummy channels to generate inter-MG crosstalk for the OAM MG under test within the same fibre core, by switching them to the associated input ports of the second OAM-MG multiplexing module. Here we note that only two branches are utilized as dummy channels due to hardware limitation and the fact that the channel crosstalk is dominated by inter-MG crosstalk. Although not all the channels are excited simultaneously, both the inter-core crosstalk and the inter-MG crosstalk within one same fibre core are simulated for each test channel in this scheme, which sufficiently resembles a transmission system with all of the 56 channels transmitted. When measurements of all the 28 OAM channels in the firstly tested OAM MG of the seven-core RCF are finished, topological charge |*l*| of the test channels and the dummy channels can be exchanged by programming the VPP diagram on the SLM in the OAM MG multiplexing module, and OAM channels in the second OAM MG can then be evaluated.

After being converted into circular polarizations, coupled into and transmitted over the 60-km seven-core RCF, the multiplexed OAM beams are collimated and converted back to linear polarization through a QWP before being divided into two branches. OAM beam in each of the two branches is converted into Gaussian beam through a VPP and then coupled into a SMF-pigtailed dual-polarization integrated coherent receiver (ICR). Only mode channels within one OAM MG in a fibre core are simultaneously de-multiplexed and received at one time and signals in different OAM MGs/fibre cores is asynchronously received. The eight output electrical waveforms (including the *I*/*Q* branches of the QPSK signal for each of the four intra-MG modes) from the ICRs are simultaneously sampled and stored by an eight-channel real-time oscilloscope operated at a sampling rate of 50 GSa/s for offline digital signal processing (DSP), which includes timing phase recovery, 4 × 4 MIMO equalization based on the conventional blind constant modulus algorithm (CMA), frequency offset compensation, and carrier phase estimation. Finally, the BERs of all four intra-MG mode channels are evaluated. This measurement is repeated for each OAM MG, each fibre core as well as each wavelength channel to evaluate the BER of all the 560 channels (7 cores × 4 OAM modes × 2 orthogonal polarizations × 10 wavelengths).

### 4.2 Results and discussion

**Figure 7:**
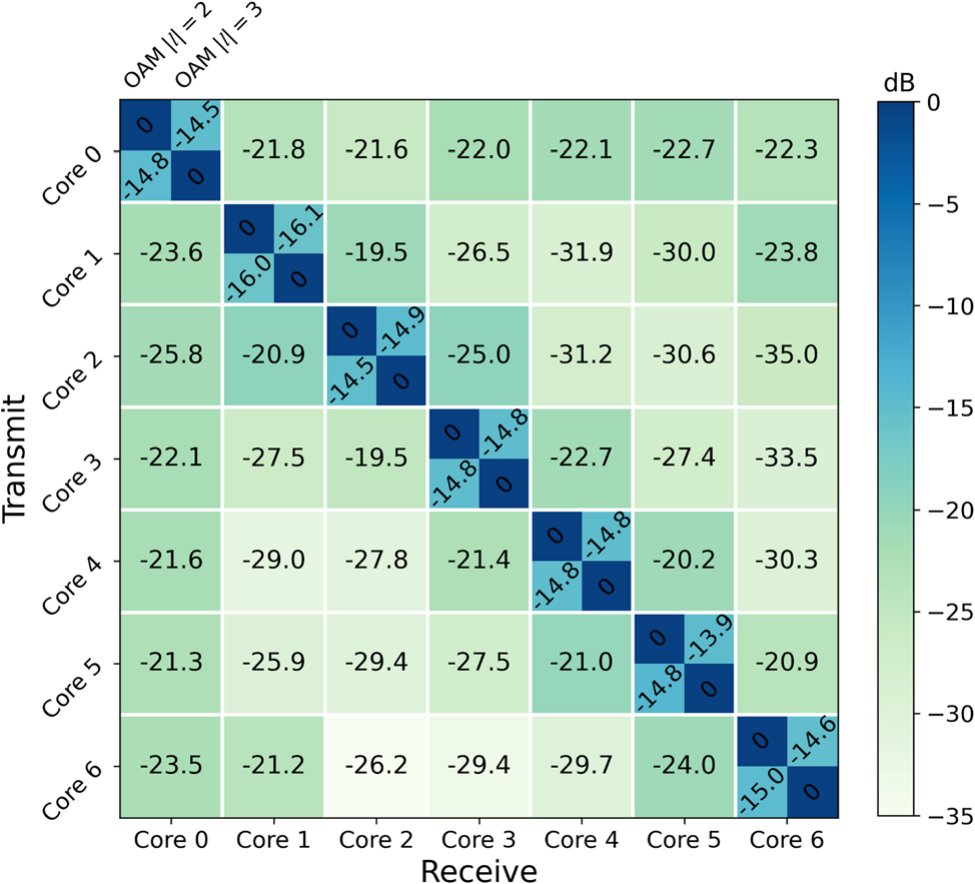
Measured system crosstalk matrix at 1550 nm.

The inter-core and inter-MG crosstalk of the subsystem including the 60-km seven-core RCF and the OAM (de)multiplexing modules (OAM mode MUX and DEMUX in the orange dashed box of Figure 6) are characterized using the power measurement method and tabulated in Figure 7. The crosstalk between OAM MGs |*l*| = 2 and |*l*| = 3 within the same fibre core is about −15 dB in average, around 8 dB higher than those between the adjacent fibre cores over the 60-km seven-core RCF. It indicates that the inter-MG crosstalk within the same fibre core dominates the transmission performance.

**Figure 8:**
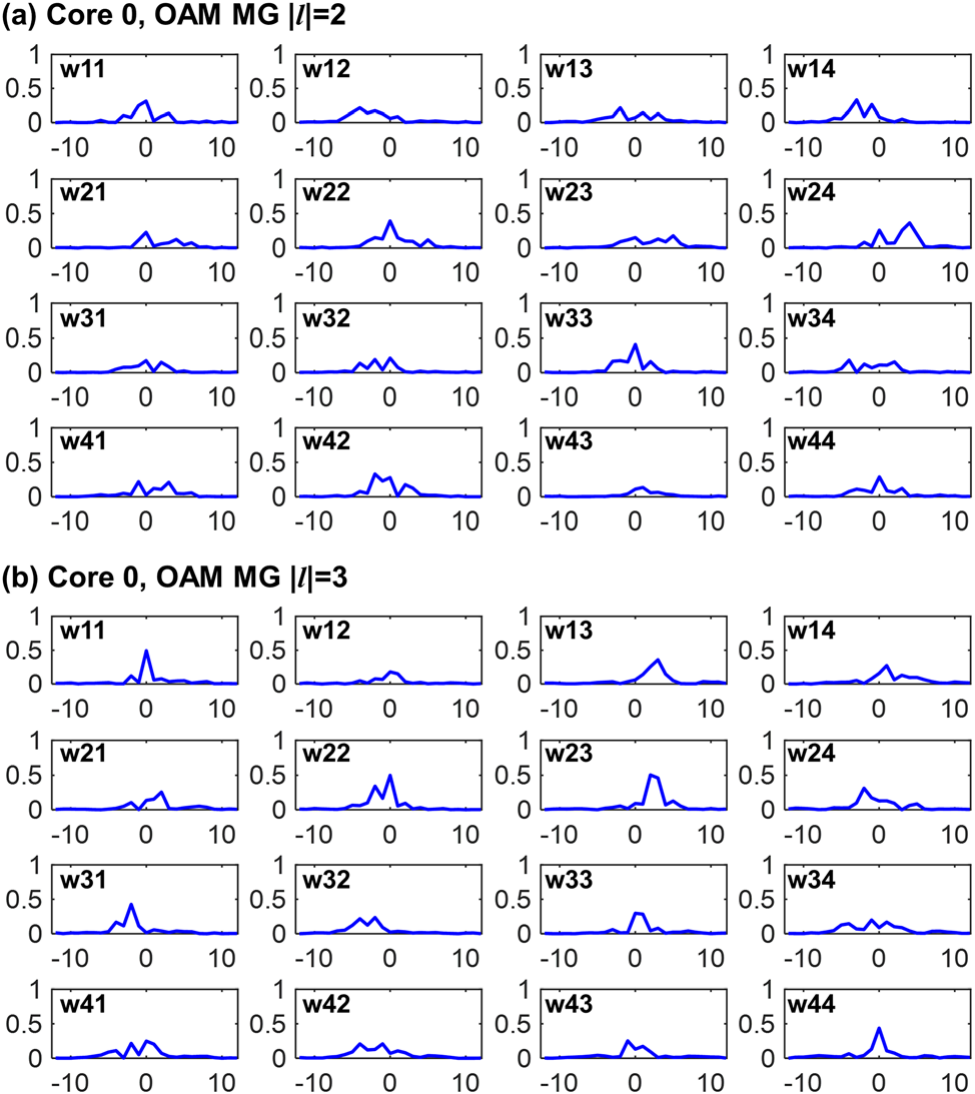
The absolute values of tap weights of 16 FIR filters in a 4×4 MIMO equalizer belonging to (a) OAM MG |*l*| = 2 and (b) OAM MG |*l*| = 3 of the central core.

Figure 8(a) and (b) illustrates the convergent tap-weight absolute values of the 16 finite impulse response (FIR) filters in the two 4 × 4 MIMO equalization modules for OAM MGs |*l*| = 2 and |*l*| = 3 in the central core [core 0 in Figure 1(a)] after 60-km transmission, which are updated using the CMA and converged after 80 iterations. The parameters of the 4 × 4 MIMO equalization modules for OAM MGs in other ring cores can be found in Supplementary S7. The number of taps in each time-domain FIR filter is set to 25, which is sufficient to cover the small differential mode delay (DMD) among intra-MG modes due to their high degeneracy. The DSP complexity could be further reduced by utilizing the frequency-domain MIMO equalization algorithm [39].

**Figure 9:**
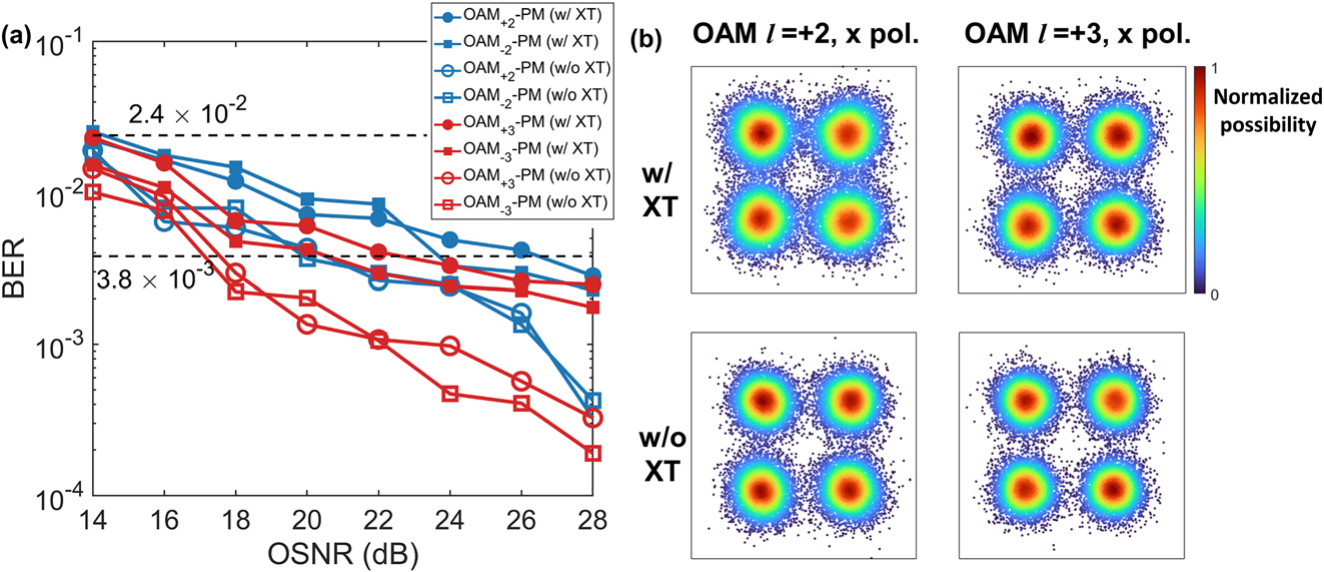
The characterization of the communication system. (a) Measured BER-OSNR curves of OAM MG |*l*| = 2 & 3 of the central core. The BER values of two orthogonal polarization states of the OAM mode with the same *l* are averaged for concision. (b) normalized probability density of the constellation points of the recovered 28-GBaud-QPSK signals belonging to OAM MG |*l*| = 2 & 3 of the central core.

In our experiments, BER measurements are carried out for both single-wavelength and WDM transmission scenarios. In the single-wavelength transmission scenario, the BER curves as a function of OSNR of OAM mode channels in the central fibre core [core 0 in Figure 1(a)] are evaluated at the wavelength of 1550.92 nm, and the BER performance with and without the inter-core and inter-MG crosstalk are compared, as shown in Figure 9. The BER values of two orthogonal polarization states of the OAM mode with the same *l* are averaged for concision. The measured BER of all the OAM modes in these two cores are below the 20% soft-decision FEC threshold of 2.4 × 10^−2^ when all the inter-core and inter-MG crosstalk exist and the OSNRs of the system is above 16 dB. Compared with the case without any inter-core/inter-MG crosstalk, the average OSNR penalty after 60-km transmission with all inter-core/inter-MG crosstalk is around 4.5 and 5 dB for the OAM MGs |*l*| = 2 and |*l*| = 3 in the central core, respectively, at the BER of 3.8 × 10^−3^ (7% hard-decision FEC threshold).

**Figure 10:**
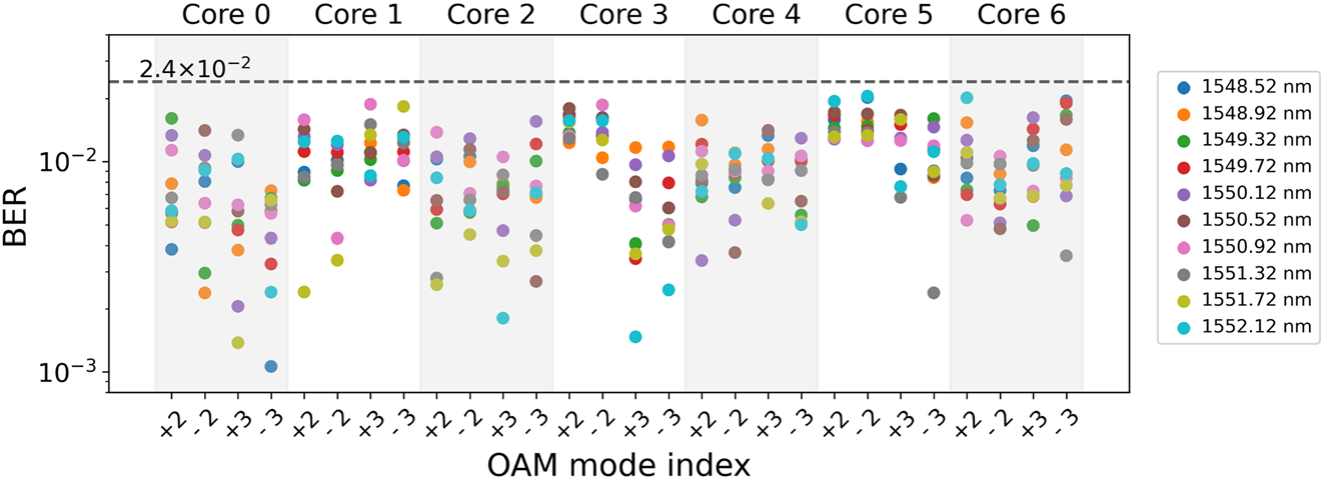
Measured BER of all 560 channels after 60 km transmission.

**Figure 11:**
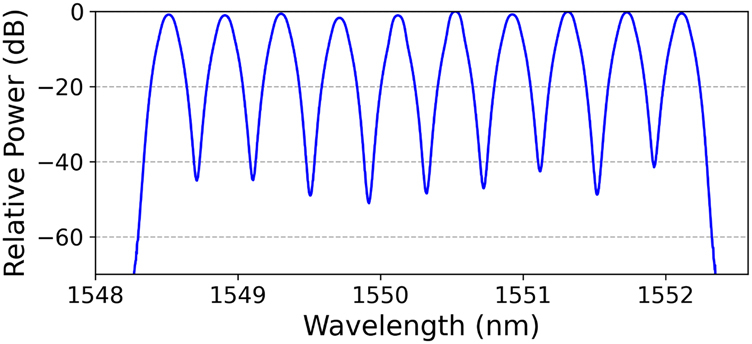
Optical spectra of the WDM signal with 10 wavelengths.

Figure 10 plots the measured BER values of all 560 channels in the WDM transmission scenario. The corresponding optical spectrum of the 10 WDM channels on the transmitter side is shown in Figure 11. BER values of all the 560 channels are below the 20% soft-decision FEC threshold of 2.4 × 10^−2^, equivalently achieving successful transmission of the data with an aggregate capacity of 31.4 Tbit/s (net 25.1 Tb/s), a SE of 62.7 bit/s/Hz (net 50.2 bit/s/Hz) and a SE-distance product of 3763.2 (bit/s/Hz) km [net 3010(bit/s/Hz) km].

## 5 Conclusions

SDM transmission based on OAM modes has been successfully demonstrated over a 60-km seven-core RCF, involving a total of 56 OAM mode channels each carrying 10 WDM channels. With measured BERs of all the 560 channels being below the 20% soft-decision FEC threshold of 2.4 × 10^−2^, an aggregate capacity of 31.4 Tbit/s (net 25.1 Tb/s), a SE of 62.7 bit/s/Hz (net 50.2 bit/s/Hz) and a SE-distance product of 3763.2 (bit/s/Hz) km [net 3010 (bit/s/Hz) km] have been achieved equivalently. Furthermore, simultaneous weak coupling among fibre cores and amongst the non-degenerate OAM MGs within each core has also been realized, so that only a modular 4 × 4 MIMO processing scheme has been needed to equalize the coupling among the near-degenerate intra-MG modes, demonstrating high scalability of the spatial channel counts and the transmission capacity while maintaining low MIMO equalization complexity and reasonable fibre cladding diameter.

## Supplementary Material

Supplementary Material

## References

[1] Richardson D. J., Fini J. M., Nelson L. E. (2013). Space-division multiplexing in optical fibres. *Nat. Photonics*.

[2] Rademacher G., Luís R. S., Puttnam B. J. (2018). 93.34 tbit/s/mode (280 tbit/s) transmission in a 3-mode graded-index few-mode fiber. *Optical Fiber Communication Conference (OFC)*.

[3] Zhang J., Liu J., Shen L. (2020). Mode-division multiplexed transmission of wavelength-division multiplexing signals over a 100-km single-span orbital angular momentum fiber. *Photonics Res.*.

[4] Turukhin A., Paskov M., Mazurczyk M. V. (2019). Demonstration of potential 130.8 Tb/s capacity in power-efficient SDM transmission over 12,700 km using hybrid micro-assembly based amplifier platform. *Optical Fiber Communication Conference (OFC)*.

[5] Kong D., Jørgensen A. A., Henriksen M. R. (2020). Single dark-pulse Kerr comb supporting 1.84 pbit/s transmission over 37-core fiber. *Conference on Lasers and Electro-Optics (CLEO)*.

[6] Rademacher G., Luís R. S., Puttnam B. J. (2021). High capacity transmission in a coupled-core three-core multi-core fiber. *J. Lightwave Technol.*.

[7] Luís R. S., Rademacher G., Puttnam B. J. (2018). 1.2 Pb/s transmission over a 160 μm cladding, 4-core, 3-mode fiber, using 368 C+L band PDM-256-QAM channels. *European Conference on Optical Communication (ECOC)*.

[8] Uden R. G. H., Correa R. A., Lopez E. A. (2014). Ultra-high-density spatial division multiplexing with a few-mode multicore fibre. *Nat. Photonics*.

[9] Shibahara K., Mizuno T., Takara H. (2016). Dense SDM (12-core × 3-mode) transmission over 527 km with 33.2-ns mode-dispersion employing low-complexity parallel MIMO frequency-domain equalization. *J. Lightwave Technol.*.

[10] Sakaguchi J., Klaus W., Mendinueta J. M. D. (2016). Large spatial channel (36-core × 3 mode) heterogeneous few-mode multicore fiber. *J. Lightwave Technol.*.

[11] Soma D., Wakayama Y., Beppu S. (2018). 10.16-Peta-B/s dense SDM/WDM transmission over 6-mode 19-core fiber across the C+L band. *J. Lightwave Technol.*.

[12] Rademacher G., Puttnam B. J., Luís R. S. (2020). 10.66 peta-bit/s transmission over a 38-core-three-mode fiber. *Optical Fiber Communication Conference (OFC)*.

[13] Mukasa K., Imamura K., Sugizaki R. (2012). Multi-core few-mode optical fibers with large Aeff. *European Conference on Optical Communication (ECOC)*.

[14] Sakamoto T., Matsui T., Saitoh K. (2017). Low-loss and low-DMD 6-mode 19-core fiber with cladding diameter of less than 250 μm. *J. Lightwave Technol.*.

[15] Liu J., Zhu G., Zhang J. (2018). Mode division multiplexing based on ring core optical fibers. *IEEE J. Quant. Electron.*.

[16] Bozinovic N., Yue Y., Ren Y. (2013). Terabit-scale orbital angular momentum mode division multiplexing in fibers. *Science*.

[17] Ung B., Vaity P., Wang L., Messaddeq Y., Rusch L. A., LaRochelle S. (2014). Few-mode fiber with inverse-parabolic graded-index profile for transmission of OAM-carrying modes. *Opt. Express*.

[18] Gregg P., Kristensen P., Ramachandran S. (2015). Conservation of orbital angular momentum in air-core optical fibers. *Optica*.

[19] Zhu L., Zhu G., Wang A. (2018). 18 km low-crosstalk OAM + WDM transmission with 224 individual channels enabled by a ring-core fiber with large high-order mode group separation. *Opt. Lett.*.

[20] Wen Y., Chremmos I., Chen Y. (2020). Compact and high-performance vortex mode sorter for multi-dimensional multiplexed fiber communication systems. *Optica*.

[21] Wang H., Yang M., Wang L. (2020). Experimental demonstration of record 300-km orbital angular momentum (OAM) mode-division multiplexing transmission using a ring-core fiber recirculating loop. *Conference on Lasers and Electro-Optics (CLEO)*.

[22] Zhu L., Li J., Zhu G. (2018). First demonstration of orbital angular momentum (OAM) distributed Raman amplifier over 18-km OAM fiber with data-carrying OAM multiplexing and wavelength-division multiplexing. *Optical Fiber Communication Conference (OFC)*.

[23] Ma J., Xia F., Chen S., Li S., Wang J. (2019). Amplification of 18 OAM modes in a ring-core erbium-doped fiber with low differential modal gain. *Opt. Express*.

[24] Berkhout G. C. G., Lavery M. P. J., Courtial J., Beijersbergen M. W., Padgett M. J. (2010). Efficient sorting of orbital angular momentum states of light. *Phys. Rev. Lett.*.

[25] Wen Y., Chremmos I., Chen Y., Zhu J., Zhang Y., Yu S. (2018). Spiral transformation for high-resolution and efficient sorting of optical vortex modes. *Phys. Rev. Lett.*.

[26] Willner A. E., Zhao Z., Liu C. (2021). Perspectives on advances in high capacity, free-space communications using multiplexing of orbital-angular-momentum beams. *APL Photonics*.

[27] Li S., Wang J. (2013). Multi-orbital-angular-momentum multi-ring fiber for high-density space-division multiplexing. *IEEE Photonics J.*.

[28] Li S., Wang J. (2014). A compact trench-assisted multi-orbital-angular-momentum multi-ring fiber for ultrahigh-density space-division multiplexing (19 rings × 22 modes). *Sci. Rep.*.

[29] Wang Y., Zhu K., Zhao W. (2021). Seven air-core fibers with germanium-doped high-index rings supporting hundreds of OAM modes. *Opt. Express*.

[30] Olshansky R. (1975). Mode coupling effects in graded-index optical fibers. *Appl. Opt.*.

[31] Jin X., Gomez A., Shi K. (2016). Mode coupling effects in ring-core fibers for space-division multiplexing systems. *J. Lightwave Technol.*.

[32] Hayashi T., Taru T., Shimakawa O., Sasaki T., Sasaoka E. (2011). Design and fabrication of ultra-low crosstalk and low-loss multi-core fiber. *Opt. Express*.

[33] Chang J. H., Bae S., Kim H., Chung Y. C. (2017). Heterogeneous 12-core 4-LP-mode fiber based on trench-assisted graded-index profile. *IEEE Photonics J.*.

[34] Maruyama R., Kuwaki N., Matsuo S., Ohashi M. (2017). Relationship between mode coupling and fiber characteristics in few-mode fibers analyzed using impulse response measurements technique. *J. Lightwave Technol.*.

[35] Born M., Wolf E. (1980). *Principles of Optics*.

[36] Feng X., Lin Z., Wen Y., Chen Y., Yu S. (2020). Arrayed vortex mode demultiplexer based on spiral transformation for dense space division multiplexing. *Asia Communications and Photonics Conference (ACP)*.

[37] Lin Z., Wen Y., Chen Y., Yu S. (2020). Transmissive multi-plane light conversion for demultiplexing orbital angular momentum modes. *Conference on Lasers and Electro-Optics (CLEO)*.

[38] Du L. B., Lowery A. J. (2012). The validity of “Odd and Even” channels for testing all-optical OFDM and Nyquist WDM long-haul fiber systems. *Opt. Express*.

[39] Arık S. Ö., Askarov D., Kahn J. M. (2014). Adaptive frequency-domain equalization in mode-division multiplexing systems. *J. Lightwave Technol.*.

