# Supplementary material for: SDM transmission of orbital angular momentum mode channels over a multi-ring-core fibre
Source: Nanophotonics. 2021 Nov 19;11(4):873–84. doi: 10.1515/nanoph-2021-0471 (PMC11501910; doi:10.1515/nanoph-2021-0471)
Supplement: Supplementary file 1 — Supplementary Material [file j_nanoph-2021-0471_suppl.docx]

Supplemental Material for: SDM Transmission of Orbital Angular Momentum Mode Channels over a Multi-ring-core Fibre

Jingxing Zhang,^1, †^ Zhongzheng Lin,^1, †^ Jie Liu,^1, *^ Junyi Liu,^1^ Zhenrui Lin,^1^ Shuqi Mo,^1^ Shuqing Lin,^1^ Lei Shen,^2^ Lei Zhang,^2^ Yujie Chen,^1^ Xiaobo Lan,^2^ and Siyuan Yu^1, *^

*^1^State Key Laboratory of Optoelectronic Materials and Technologies, School of Electronics and Information Technology, Sun Yat-sen University, Guangzhou 510006, China*

*^2^State Key Laboratory of Optical Fiber and Cable Manufacture Technology, Yangtze Optical Fiber and Cable Joint Stock Limited Company, Wuhan 430073, China*

*† These authors contributed equally*

**Corresponding author:* [*liujie47@mail.sysu.edu.cn*](mailto:liujie47@mail.sysu.edu.cn)*;* [*yusy@mail.sysu.edu.cn*](mailto:yusy@mail.sysu.edu.cn)*.*

**S1. Calculation of the inter-core crosstalk (ICXT)**

**S2. Measurement of the fibre propagation loss**

**S3. Measurement of the differential mode-group delay (DGD)**

**S4. Deduction of the coupling principle of the ‘7-to-7’ scheme**

**S5. Details of the beam size and phase matching**

**S6. Robustness analysis for the ‘7-to-7’ coupling scheme**

**S7. Absolute tap-weight values of all spatial channels**

**S1. Calculation of the inter-core crosstalk (ICXT)**

For MCF with identical cores, the mean crosstalk between adjacent cores can be estimated using the following expression [1],

$${XT}_{mean}=\frac{2\kappa^{2}R}{\beta\Lambda}L$$

where $\beta$ is the propagation constant of selected mode, $\Lambda$($50um$) and $L$ denotes the core pitch and fibre length respectively, $R$ is the bending radius of fibre ($140mm$). $\kappa$ is the mode coupling coefficient which can be expressed between core $n$ and $m$ as follows [2]，

$$k_{nm}=\frac{\omega\varepsilon_{0}\int_{-\infty}^{+\infty} \int_{-\infty}^{+\infty} (N^{2}-N_{m}^{2})\boldsymbol{E}_{\boldsymbol{n}}^{\boldsymbol{*}}\boldsymbol{\cdot}\boldsymbol{E}_{\boldsymbol{m}}dxdy}{\int_{-\infty}^{+\infty} \int_{-\infty}^{+\infty} \boldsymbol{e}_{z}\cdot(\boldsymbol{E}_{\boldsymbol{n}}^{\boldsymbol{*}}\boldsymbol{\times}\boldsymbol{H}_{\boldsymbol{n}}\boldsymbol{+}\boldsymbol{E}_{\boldsymbol{n}}\boldsymbol{\times}\boldsymbol{H}_{\boldsymbol{n}}^{\boldsymbol{*}})dxdy}$$

where $\omega$ is the angular frequency of the optical signal, $\varepsilon_{0}$ is the permittivity of vacuum, $N^{2}(x,y)$ and $N_{m}^{2}$ represent the refractive index distribution of the entire MCF and the refractive index distribution of waveguide $m$ (which includes core $m$ and cladding in MCF) respectively. $\boldsymbol{E}$ and $\boldsymbol{H}$ are the normalized electric field and magnetic field of the selected modes, $\boldsymbol{*}$ denotes the complex conjugate, $\boldsymbol{e}_{z}$ is the unit vector in the direction of propagation. The calculation results of ICXT after 100-km propagation is shown in Fig. S1


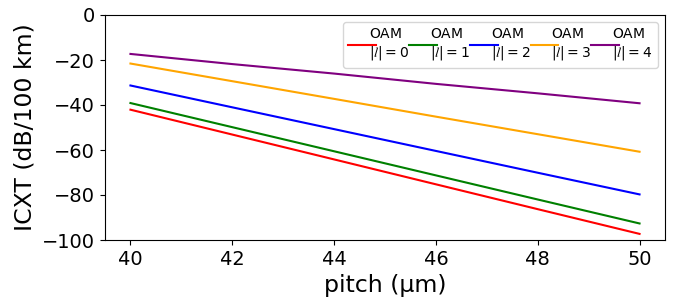


Fig. S1 The calculated ICXT after 100-km propagation versus core pitch at 1550nm.

S2. Measurement of the fibre propagation loss

The propagation loss of the fabricated 7-core RCF is measured by the optical time domain reflectometer (OTDR) method. As illustrated Fig. S2, the signal from the OTDR is converted to an OAM mode by VPP and then couples to one core of the 7-core RCF. OAM modes of different orders are selectively excited using different VPP in turn. The same measurement process is repeated for each core.


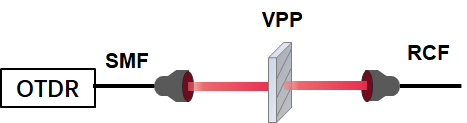


Fig. S2 Experimental setup for measuring the fibre propagation loss.


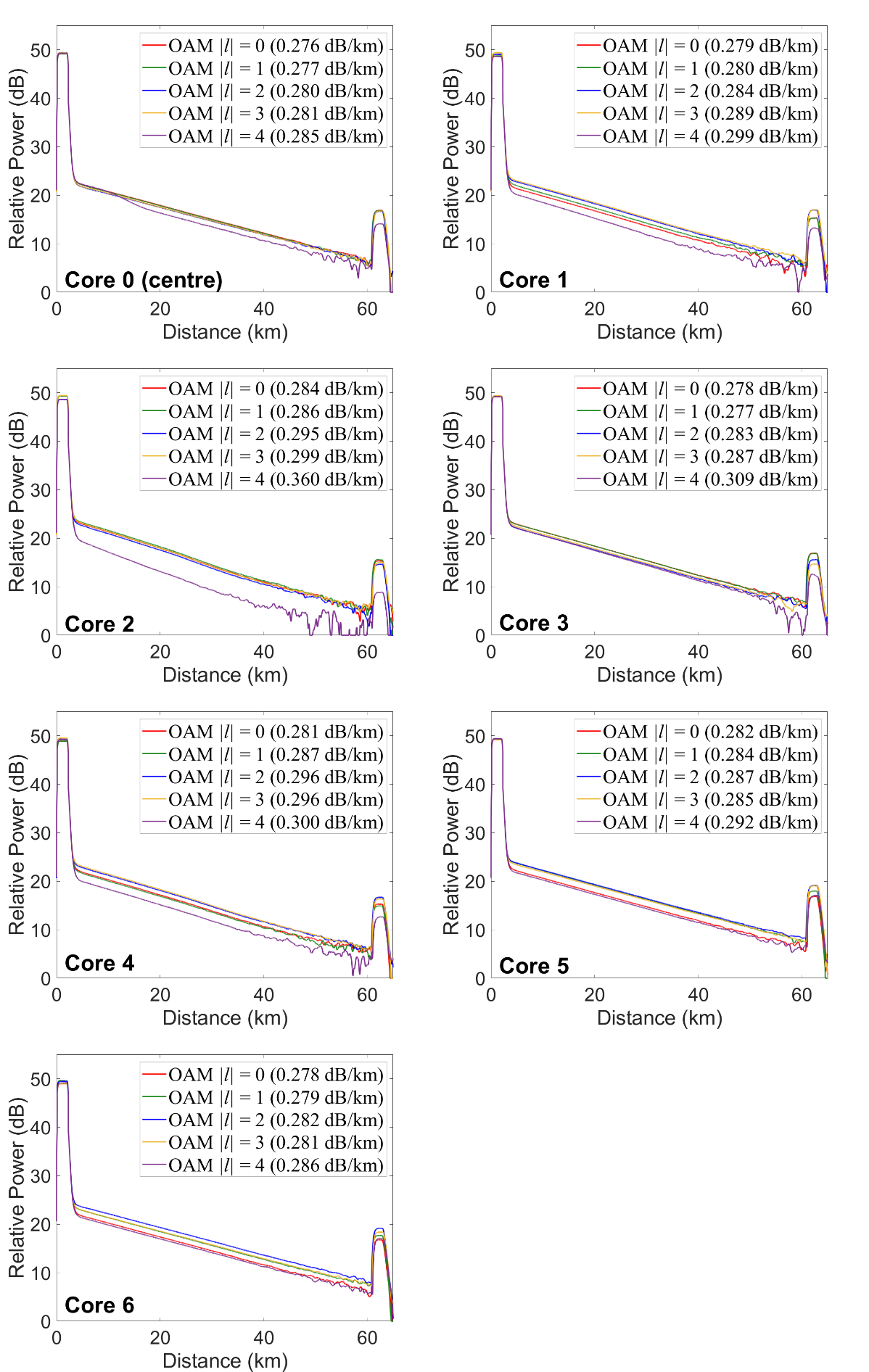


Fig. S3 Attenuation measured by the OTDR.

S3. Measurement of the differential mode-group delay (DGD)

We utilize the vector network analyzer (VNA) to measure the differential mode-group delay (DGD) for each core. The experimental setup is illustrated Fig. S4. the VNA generates swept-frequency radio frequency (RF) signals that drive the Mach-Zehnder modulator (MZM). A narrow-linewidth laser at 1550 nm is modulated in the Mach-Zehnder modulator (MZM), amplified by the erbium doped fibre amplifier (EDFA), and then collimated to a free space beam. The collimated beam is polarization managed by a linear polarizer to match the working axis of the spatial light modulator (SLM). A vortex phase plate (VPP) is loaded on the SLM and convert the collimated Gaussian beam into an OAM mode. The OAM beam is then converted to circular-polarization by a quarter-wave plate (QWP) and focused into one core of the 7-core ring-core fibre. After transmission through the 60 km fibre span, the beam is detected by a photo detector (PD) with a multi-mode fibre (MMF) pigtail. Finally, the detected RF signals are fed back to the VNA and the impulse response is measured by setting the VNA to the impulse response mode. The same measurement process is repeated for each core. The DGD is determined by the relative delay between the different peaks that represent the modes (or mode groups) of the fibre core.


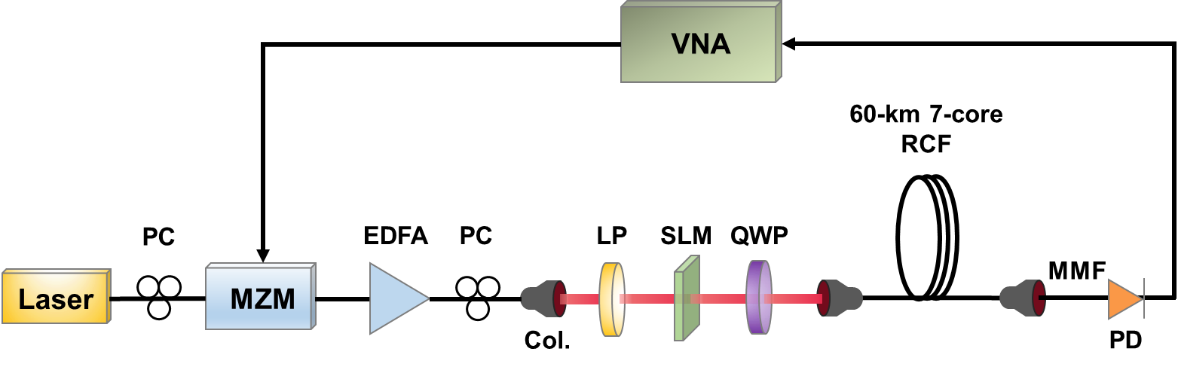


Fig. S4 VNA based impulse response measurement setup for determining mode coupling in a fibre. VNA: vector network analyzer; PC: polarization controller; MZM: Mach-Zehnder modulator; EDFA: erbium doped fibre amplifier; Col.: collimator; LP: linear polarizer; SLM: spatial light modulator; QWP: quarter-wave plate; PD: photo detector.

**S4 Deduction of the coupling principle of the ‘7-to-7’ scheme**

As described in the main text, we use a single phase-only spatial modulation to realize coupling from a 7-core SMF to the 7-core RCF. We first generally discuss how to achieve maximum coupling efficiency through single phase-only modulation, then apply it to ‘7-to-7’ coupling scheme.

On the plane of the phase mask, we aim to convert mode into with the phase-only modulation . Assume $u_{f}$and $u_{b}$ is normalized in power, the conversion efficiency is [3]

(1)

As we can always make the phase modulation to perfectly compensate the phase mismatch, the conversion efficiency becomes

$\eta=\left|\iint A_{f}A_{b}dxdy\right|^{2}$ (2)

Equation 2 indicates that the achievable maximum conversion efficiency depends on the overlap of$A_{f}$and $A_{b}$. As $A_{f}$ and $A_{b}$ vary with the relative position and rotation of the elements in the ‘7-to-7’ scheme, the principle that maximizing the coupling efficiency in the ‘7-to-7’ scheme can be deduced as below:

1) the real amplitude distribution (practically intensity distribution in experiment as can be directly observed) of the forward propagating beams from the 7-core SMF and the backward propagating beams from the 7-core RCF should maximally overlap on the phase mask;

2) the phase mask should perfectly compensate the phase mismatch between the forward and backward propagating beams.

**S5. Details of the beam size and phase matching**


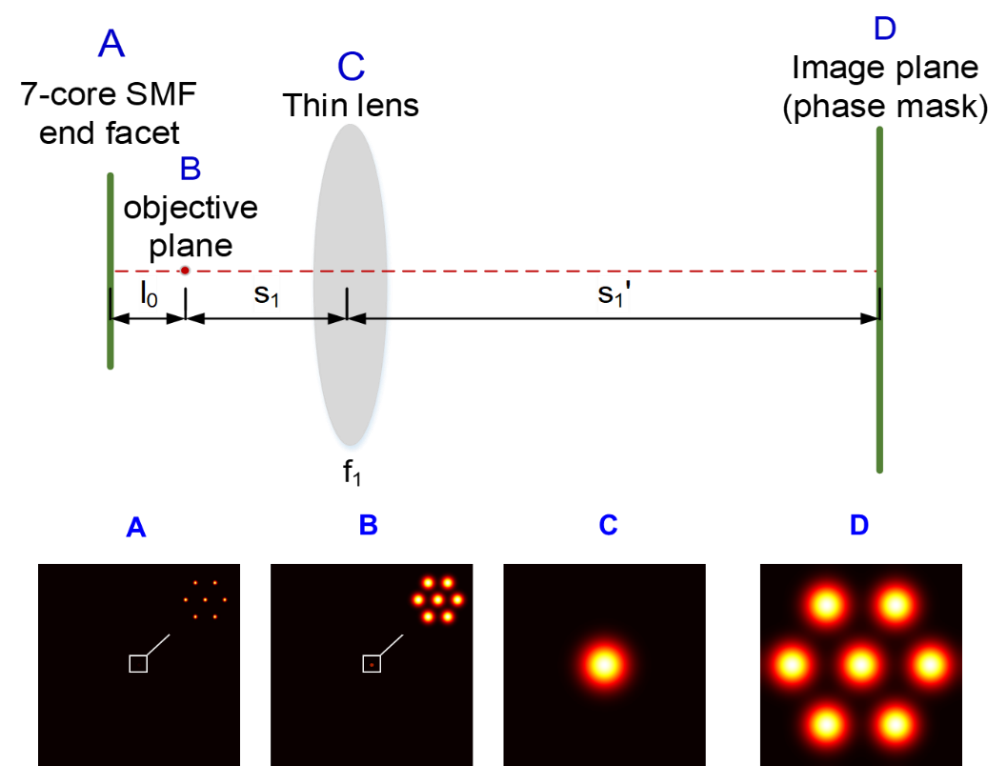


Fig. S5 Schematic of increasing the ratio of beam diameter to core pitch on the image plane by deviating 7-core SMF end face far from the objective plane.

In the main text, it is described that we simply imaging the end facet of the 7-core SMF and 7-core RCF to the plane of the phase mask and keep the pitches the same on the plane. However, the beam size will not be best-matched in this way as the ratio of beam diameter to core pitch of the 7-core SMF is smaller than that of the 7-core RCF, in other words, the Gaussian beam would be smaller than the OAM mode on the phase mask, which would deteriorate the coupling efficiency.

To increase the Gaussian beam size while maintaining the pitch on the phase mask, we make the 7-core SMF slightly deviate (about 40μm in our experiment) from the objective plane of the lens. As illustrated in Fig. S5, during the free-space propagating from the end facet (plane A) to the objective plane (plane B), the 7 Gaussian beams from 7-core SMF will diverge. Each Gaussian beam becomes wider but with the pitch unchanged, as such the ratio of beam diameter to core pitch is increased on the objective plane. The beam profile on the objective plane is then imaging to the phase mask (plane D) with a scaling factor $V_{i}$.

With the real amplitude distribution best-matched on the phase mask, the maximal coupling efficiency can be achieved by compensate all the phase mismatch on that plane. The phase mismatch containing not only the azimuthal vortex phase around each beam axis, but also the radial quadratic phase induced by lens imaging around the global optical axis. The radial quadratic phase can be cancelled by a Fresnel lens with focal length of $V_{i}f_{i}$, where$f_{i}$ represents the focal distance of the thin lens. Subscript *i* equals to 1 or 2, denotes parameters on the 7-core SMF side or 7-core RCF side. Therefore, a Fresnel lens should superimpose on the vortex phase to match the quadratic phase, and the focal distance of the Fresnel lens satisfies [4]

$\frac{1}{f}=\frac{1}{V_{1}f_{1}}+\frac{1}{V_{2}f_{2}}$ (3)

where *f* represents the equivalent focal distance of the Fresnel lens and is calculated to be 226mm in our experiment, with the parameters shown in Table 3.

**S6. Robustness analysis for the ‘7-to-7’ coupling scheme**

Due to errors in the optical path or the device, the beams from 7-core SMF and 7-core RCF may not overlap perfectly on the phase mask. We analyse extra loss due to rotation, pitch, and beam size errors by calculating$\eta=\left|\iint u_{f}^{'}\Phi u_{b}^{*}dxdy\right|^{2}$, where $u_{f}^{'}$ and $u_{b}$ represents forward propagating beams from the 7-core SMF (with some errors) and the backward propagating beams from the 7-core, respectively. represents the phase distribution of the phase mask. It is assumed that the error only occurs on the 7-core SMF side. Note that the rotation and pitch error only affect the coupling efficiency of the outer cores.


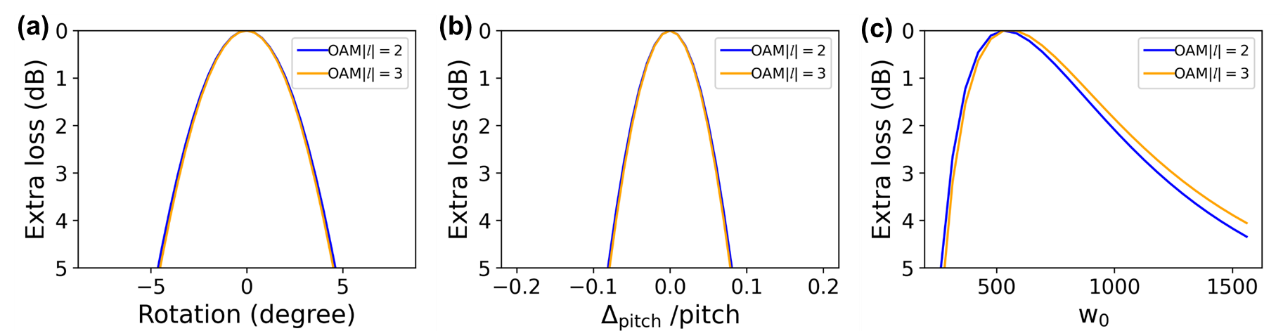


Fig S6. Robustness analysis for the ‘7-to-7’ coupling scheme. (a) Extra loss induced by rotation error. (b) Extra loss induced by pitch error. (c) Extra loss induced by beam size error on the phase mask. The experiment setup is assumed the same as those listed in Table 3 and the phase mask is assumed always perfectly compensate the phase mismatch.

S7. Absolute tap-weight values of all spatial channels

The absolute values of complex tap weights of all spatial channels in all outer cores at 1550.92 nm are shown below. As the tap-weight absolute values of the centre core have already listed in the main text, here we only list the values of the 6 outer cores. The constant modulus algorithm (CMA) iterates for 80 times, and the number of taps of each FIR filter is set to be 25.


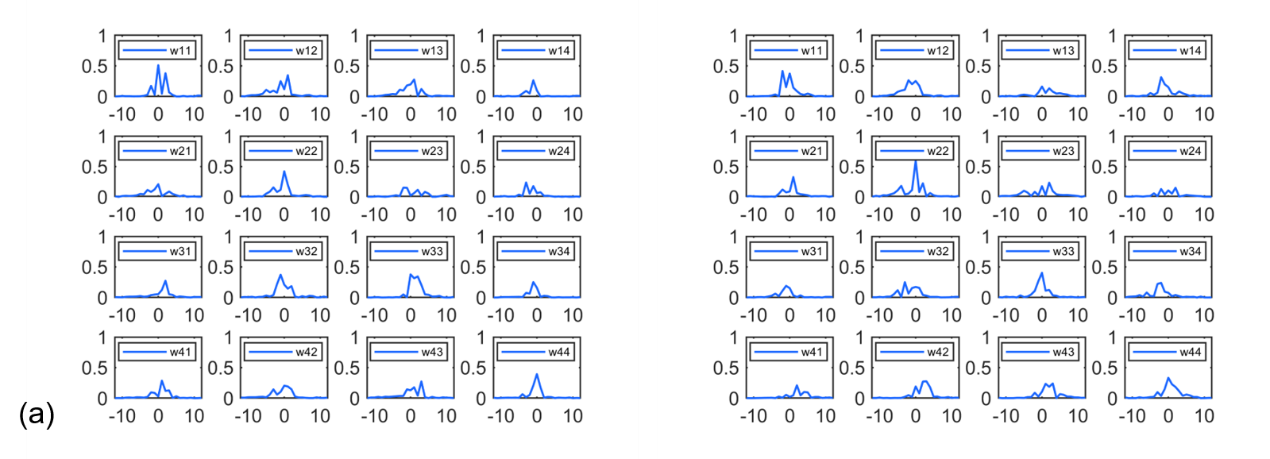


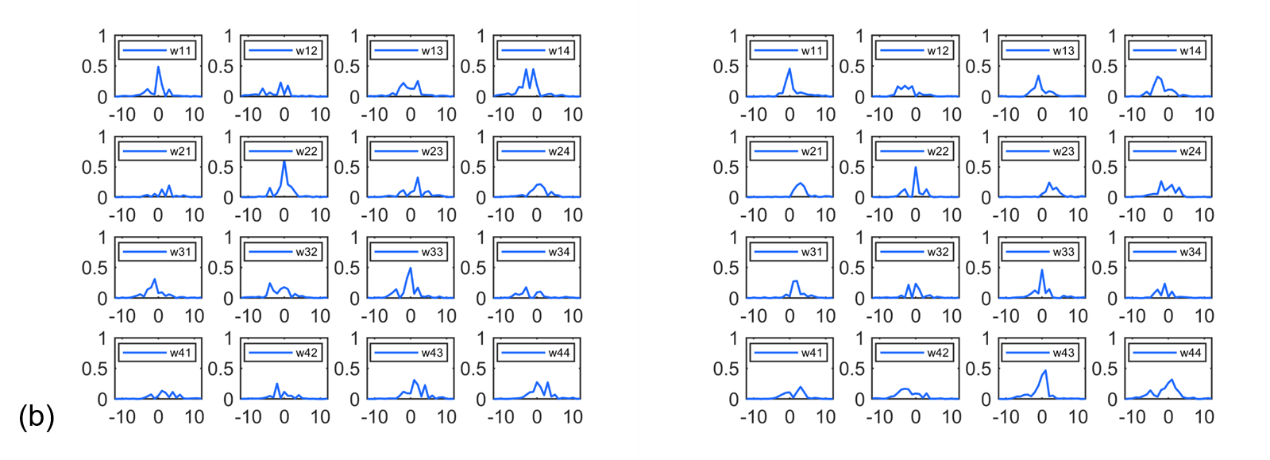


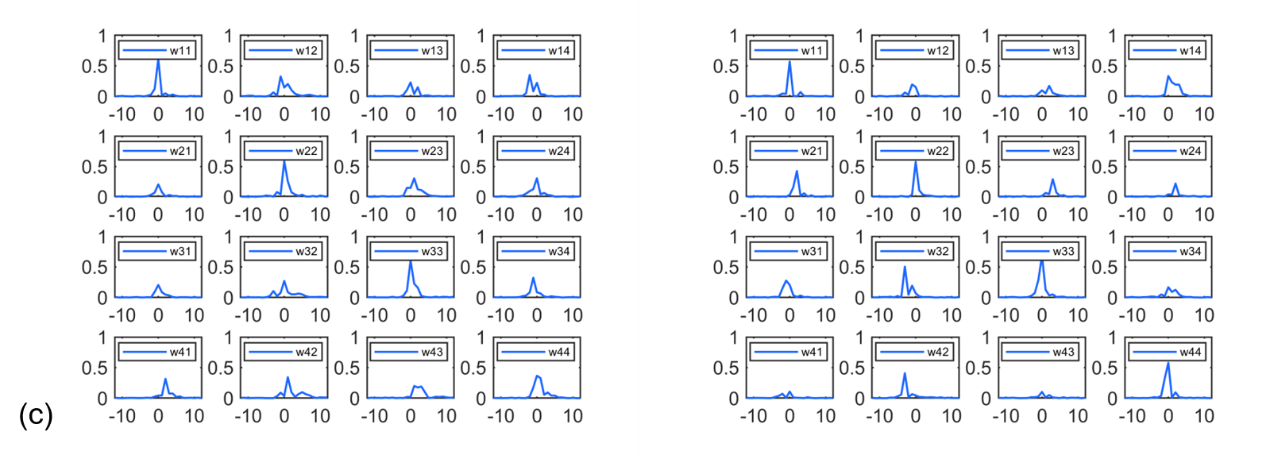


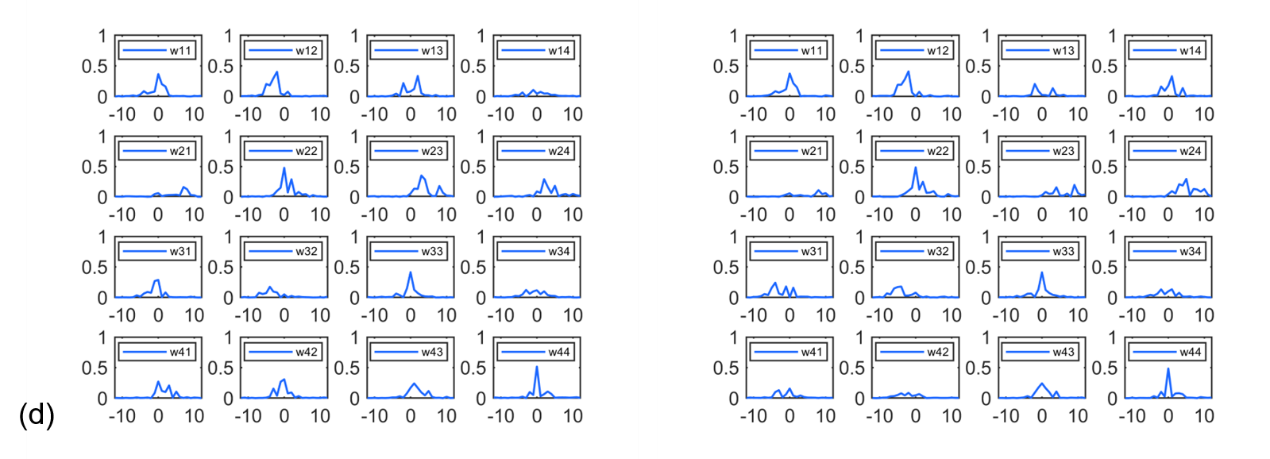


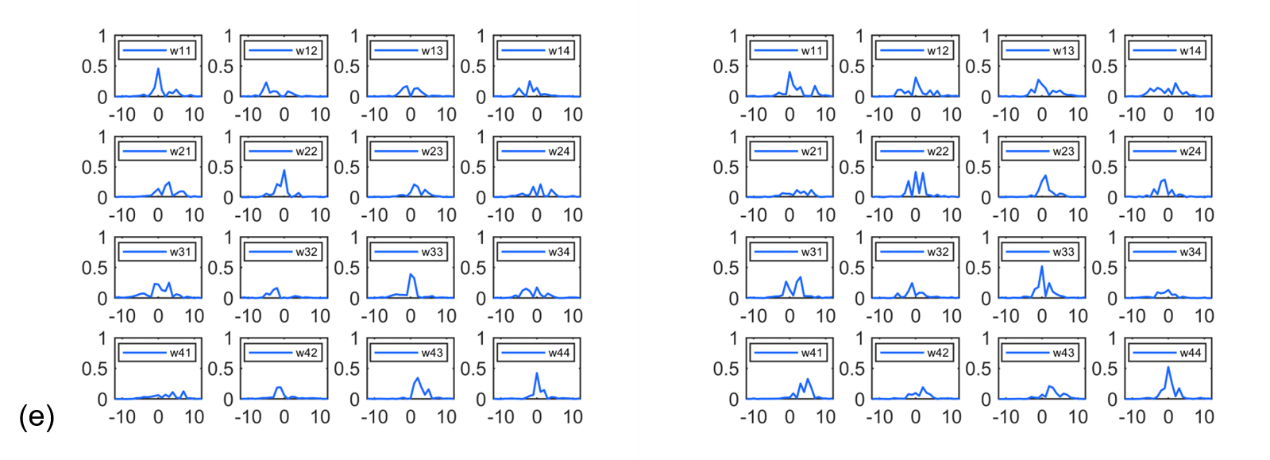


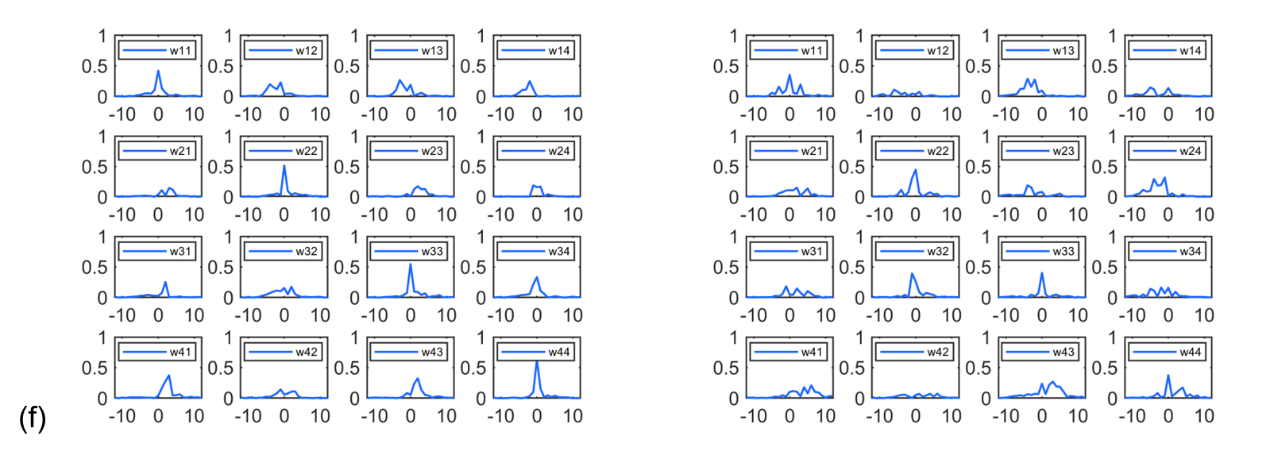


Fig. S7 The absolute values of tap weights of 4x4 MIMO equalizer of all spatial channels. (a) core 1, (b) core 2, (c) core 3, (d) core 4, (e) core 5 and (f) core 6. The subplots on the left represents OAM MG |*l*|=2, and the subplots on the right represents OAM MG |*l*|=3.

**Reference**

[1] T. Hayashi, T. Taru, O. Shimakawa, T. Sasaki, and E. Sasaoka, "Design and fabrication of ultra-low crosstalk and low-loss multi-core fiber," *Opt*. *Express*, vol. 19, pp. 16576-16592, 2011.

[2] K. Okamoto, “Fundamentals of Optical Waveguides, 2nd edition” (Academic Press, 2006), pp. 159-187.

[3] J.–F. Morizur, L. Nicholls, P. Jian, et al., “Programmable unitary spatial mode manipulation,” *J. Opt. Soc. Am. A*, vol. **27**, pp. 2524-2531, 2010.

[4] M. Born and E. Wolf, “Principles of Optics, 5th edition” (Cambridge University Press,1980), pp. 157-164.
